# Bispecific NKG2D-CD3 and NKG2D-CD16 fusion proteins for induction of NK and T cell reactivity against acute myeloid leukemia

**DOI:** 10.1186/s40425-019-0606-0

**Published:** 2019-05-29

**Authors:** Melanie Märklin, Ilona Hagelstein, Samuel P. Koerner, Kathrin Rothfelder, Martin S. Pfluegler, Andreas Schumacher, Ludger Grosse-Hovest, Gundram Jung, Helmut R. Salih

**Affiliations:** 1 Clinical Collaboration Unit Translational Immunology, German Cancer Consortium (DKTK) and German Cancer Research Center (DKFZ), Partner site Tuebingen, Otfried-Mueller-Str. 10, 72076 Tuebingen, Germany; 2 Department for Immunology, Eberhard Karls University, Tuebingen, Germany

**Keywords:** AML, NKG2DL, Bispecific, CD3, CD16, Leukemia, mAb, Fc-optimized, Fusion protein

## Abstract

**Background:**

Monoclonal antibodies (mAbs) mediate their effects in great part by inducing ADCC of NK cells, and multiple efforts aim to increase this function by engineering mAbs optimized Fc-parts. Even more potent antitumor immunity can be induced by strategies to stimulate T cells with their profoundly higher effector potential. However, upon increased immunostimulatory potential, the necessity to target highly tumor-specific antigens becomes critically important to reduce side effects.

**Methods:**

We here report on bispecific fusion proteins (BFP) that target ligands of the immunoreceptor NKG2D (NKG2DL), which are widely expressed on malignant cells but generally absent on healthy tissue. They consist of the extracellular domain of NKG2D as targeting moiety fused to Fab-fragments of CD3 (NKG2D-CD3) or CD16 (NKG2D-CD16) antibodies.

**Results:**

NKG2D-CD16 displayed increased affinity to the FcγRIII on NK cells compared to engineered Fc-parts, which are contained in optimized mAbs that presently undergo clinical evaluation. In line, NKG2D-CD16 induced superior activation, degranulation, IFN-γ production and lysis of acute myeloid leukemia (AML) cell lines and patient AML cells. NKG2D-CD3 in turn potently stimulated T cells, and comparison of efficacy over time revealed that NKG2D-CD16 was superior upon short term application, while NKG2D-CD3 mediated overall more potent effects which manifested after longer times. This can be attributed to treatment-induced proliferation of T cells but not NK cells.

**Conclusions:**

Taken together, we here introduce novel “antibody-like” BFP that take advantage of the highly tumor-restricted expression of NKG2DL and potently activate the reactivity of NK cells or T cells for immunotherapy of AML.

**Electronic supplementary material:**

The online version of this article (10.1186/s40425-019-0606-0) contains supplementary material, which is available to authorized users.

## Background

In many cancer entities, the introduction of monoclonal antibodies (mAbs) has significantly improved the treatment options for patients. This is exemplified by Rituximab and Herceptin, the first antitumor antibodies that became clinically available and meanwhile have become a mainstay of therapy in patients with B cell Non-Hodgkin’s lymphoma and Her2-positive breast cancer, respectively [1, 2]. Nevertheless, the success of antibodies in cancer treatment has its limitations: First, for many cancer entities including acute myeloid leukemia (AML), which is at the focus of this study, so far no approved immunotherapeutic antibodies are available. Second and maybe more importantly, the efficacy of currently available anti-tumor mAbs is far from being satisfactory.

To overcome the first limitation, identification of novel target antigens that are expressed broadly in a given cancer entity while being absent on healthy tissues is required. Following this reasoning, we recently introduced a construct termed NKG2D-Fc-ADCC that consists of the extracellular domain of the immunoreceptor NKG2D fused to an optimized Fc-part (amino acid modifications S239D and I332E (SDIE)) to induce antibody dependent cellular cytotoxicity (ADCC) of NK cells [3]. The latter is an important function by which monoclonal antibodies mediate their beneficial effects, in particular in hematological malignancies [3, 4]. NKG2D recognizes various ligands (NKG2DL) of the MHC class I-related chain (MIC) and UL16-binding protein (ULBP) family of proteins that are selectively overexpressed on malignant cells including leukemia, but largely absent on healthy tissues [5, 6]. Utilizing the NKG2D receptor as targeting moiety allowed to simultaneously bind all different NKG2DL with their highly variable expression pattern on malignant cells of different patients [5, 6]. We found that NKG2D-Fc-ADCC mediates potent antitumor efficacy in both, solid tumors and leukemia including AML [7, 8]. Nevertheless, the efficacy of NKG2D-Fc-ADCC still leaves room for improvement. The SDIE modification contained in the engineered Fc part largely increases the affinity to CD16, but NKG2D-Fc-ADCC may bind to inhibitory FcγR. This in turn may decrease its immunostimulatory potential. In addition, NKG2D-Fc-ADCC does not allow for stimulation of T cells. In fact, it is T cell-recruiting strategies that recently have revolutionized cancer treatment, at least in some entities. Beyond immune checkpoint inhibition and chimeric antigen receptor (CAR) T cells, this encompasses bispecific antibodies (bsAb) like Blinatumumab (Amgen), the prototypical BiTE bsAb with CD19×CD3 specificity approved for treatment of acute lymphoblastic leukemia (ALL) [9, 10]. However, sustained therapeutic success of so far available T cell-mobilizing strategies in general is forestalled by severe side effects that are caused by unspecific activation of the T cell system, which particularly holds true for Blinatumomab. This limits safely applicable doses, which in turn prevents therapeutically optimal dosing resulting in loss of efficacy. Among others, this is due to the fact that many employed target antigens are not only expressed on malignant, but also on healthy cells, such as B cells in case of CD19/Blinatumomab.

Based on the aforementioned considerations, we reasoned that refining our previous concept to target NKG2DL by improving the immunostimulatory moiety of the constructs would allow for a still highly tumor specific immunotherapeutic strategy with improved efficacy. To this end, we conceptualized NKG2D-CD16 and NKG2D-CD3 bispecific fusion proteins (BFP) with effector parts consisting of anti-CD16 and anti-CD3 Fab-fragments, respectively. The first would bind CD16 specifically and with increased affinity, while the second would allow for recruitment of T cells. In the present study, these novel BFP were preclinically characterized using AML cell lines and primary cells of AML patients in functional analyses with human NK cells and T cells.

## Methods

### PBMC and cell lines

Peripheral blood mononuclear cells (PBMC) of patients and healthy donors were isolated by density gradient centrifugation. The human cell lines THP-1, MOLM-14 and NB-4 were obtained from DSMZ (Braunschweig, Germany). The C1R-MICA transfectants were described previously [6]. Authenticity of cell lines was determined by single nucleotide profiling, and the respective immunophenotype described by the provider was further validated using FACS every 6 month and specifically prior to use in experiments. Mycoplasma contamination was excluded by routine testing of cell lines every 3 months.

### Production and purification of NKG2D fusion proteins

The NKG2D-Fc-ADCC fusion protein was generated as described previously [7, 8]. To generate NKG2D-CD3 and NKG2D-CD16 BFP, the extracellular domain of NKG2D (F78-V216) was fused using a CH_2_-linker C-terminally to a heavy chain of a Fab-fragment specific either for CD3 (clone UCHT1) or CD16 (clone 3G8), respectively [11, 12]. The CH_2_-domain of IgG1 was attenuated for FcγR binding, complement fixation and to prevent binding to glycan receptors and reduction of immunogenicity. The following amino acids were exchanged or deleted: E233→P; L234→V; L235→A; G236→deleted; D265→G; N297→Q; A327→Q; A330→S. The modification N297→Q prevents the addition of a glycan structure and Cys226 and Cys229 were exchanged to Ser to prevent dimerization. SP2/0-Ag14 cells (American Type Culture Collection, Manassas) were co-transfected with vectors coding for the different constructs together with vectors coding for the respective light chain of the Fab-fragment by electroporation. Subcloned transfectants were cultured in IMDM supplemented with 1 mg/mL G418. BFP were purified from culture supernatants by HiTrap KappaSelect™ affinity chromatography (GE Healthcare, Munich, Germany) followed by preparative size exclusion chromatography on Superdex HiLoad 16/60 column (GE Healthcare). Purity was determined by 4–12% gradient non-reducing SDS-PAGE and analytical size exclusion chromatography using a Superdex 200R PC3.2/30 column (GE Healthcare). The endotoxin (EU) levels were ≤ 1 EU/mg for all proteins.

### Flow cytometry

Flow cytometry was conducted using unconjugated NKG2D-CD3, NKG2D-CD16 or a recombinant NKG2D-Fc chimera (fusion protein) (R&D, Minneapolis, MN), for simultaneous staining of all NKG2DL. NKG2D-Fc chimera and the corresponding isotype control were biotinylated with the One-step biotinylation kit (MACS Miltenyi, Bergisch Gladbach, Germany) according to manufacturer’s instructions. Cells were blocked with human or mouse IgG (Sigma-Aldrich, St. Louis, MO) prior to staining then washed and followed by adding NKG2D-CD3, NKG2D-CD16 or biotinylated NKG2D-Fc chimera or control (10 μg/mL each) detected with streptavidin-PE or donkey-anti-human-PE (LifeTechnologies, Carlsbad, CA). Fluorescence-conjugates (CD3, CD4, CD8, CD25, CD33, CD34, CD38, CD117, CD56, CD69 and CD107a), all from BioLegend, San Diego, CA) were used in 1:100–1:200 dilutions. Dead cells were excluded from analysis with 7-AAD (BioLegend) or LIVE/DEAD™ Fixable Aqua (Thermo Fisher Scientific, Waltham, MA). Fixation of stained cells was done with 2% paraformaldehyde (Sigma-Aldrich).

FACS-based determination of antibody-induced target lysis was conducted as follows: In the allogeneic setting, leukemic cells were loaded with 5 μM CellTrace™ Violet cell proliferation dye (Thermo Fisher Scientific, Waltham, MA) and cultured with PBMC of healthy donors in the presence or absence of the constructs (10 μg/mL each). Dying and dead AML cells were identified based on propidium iodide (PI) (Sigma-Aldrich) positivity. In the autologous setting, constructs (10 μg/mL each) were directly added to PBMC of AML patients with moderate blast counts (26–75%). Then AML cells were identified according to the immunophenotype obtained at diagnosis (staining for CD33, CD34, CD38 and/or CD117) and dying and dead cells were detected based on PI (Sigma-Aldrich) positivity. In both settings, analysis of equal assay volumes was ascertained by using standard calibration beads, which allowed accounting for the number of target cells that had vanished from the culture. The percentage of living target cells was calculated as follows: PI- cells upon treatment/PI-negative cells in control × 100.

Specific fluorescence indices (SFIs) were calculated by dividing median fluorescences obtained with specific monoclonal antibodies by median fluorescences obtained with isotype control. Expression was considered positive in case of SFI ≥1.5. Measurements were conducted using a FACSCanto II or a LSR Fortessa (BD Biosciences, Heidelberg, Germany) and data analysis was performed using FlowJo software (FlowJo LCC, Ashland, OR).

### Analysis of NK and T cell activation and degranulation

To determine activation and degranulation in the absence of target cells, 10 μg/mL anti-NKG2D mAb (6H7) [6] was coated on 96-well plates overnight and washed, followed by 2 h incubation with the NKG2D fusion proteins or controls (10 μg/mL each). Subsequently plates were washed and 1 × 10^6^ PBMC of healthy donors were added followed by flow cytometry.

To determine activation and degranulation in the presence of target cells, 20,000 primary AML cells were cocultured with allogenic PBMC of healthy donors (E:T ratio 2.5:1) followed by flow cytometry.

### Analysis of expression and secretion of granzyme and perforin

PBMC were cultured with immobilized BFP in the absence of target cells or with patient AML cells for 24 h. Monensin (GolgiStop, BD Biosciences) was added 8 h prior to flow cytometric analysis. Intracellular flow cytometry was conducted using the Cytofix/Cytoperm Fixation/Permeabilization Solution Kit (BD Biosciences) according the manufacturer’s instructions. For detection of intracellular proteins, fluorescence-conjugated granzyme B and perforin antibodies (both from BioLegend) were used in 1:25 dilutions. For assessment of cytokine secretion, PBMC were treated as described above but without adding GolgiStop to allow for analyses of cytokine secretion into supernatants by Legendplex assays (BioLegend).

### Cytotoxicity assay

Lysis of cell lines and primary AML cells by allogenic PBMC was analyzed by 2 h BATDA Europium assays as described previously [8]. Percentage of lysis was calculated as follows: 100 × (experimental release - spontaneous release)/(maximum release - spontaneous release). Long-term cytotoxicity experiments were performed using the IncuCyte® S3 Live-Cell Analysis System (Essenbioscience, Sartorius, Göttingen). THP-1 cells (5000 cells/well) were labeled with 0.5 μM CytoLight Rapid Red reagent according to the manufacturer’s protocol and seeded in poly-L-ornithine (Sigma-Aldrich) coated 96-well plates with PBMC of healthy donors (E:T 20:1) in the presence of 250 nM Cytotox Green Reagent to detect dead cells. The different constructs and controls were added as indicated (all at 10 μg/mL). Live cell imaging pictures were taken every 2 h with the 4× magnification.

### Determination of IFN-γ

Interferon-γ (IFN-γ) levels in culture supernatants were analyzed by ELISA using the ELISA mAb set from Thermo Scientific (Waltham MA), according to manufacturer’s instructions. All indicated concentrations are expressed as means of triplicate measurements with standard error of the mean (SEM).

### T cell proliferation

PBMC (2 × 10^5^) were seeded in triplicates in 96-well plates with varying concentrations of the BFP and with irradiated (100 Gy) NKG2DL-positive MOLM-14 cells (0.5 × 10^5^). After incubating for 48 h, cells were pulsed with ^3^H-methyl-thymidine (0.5 μCi/well) for 20 h and harvested on filtermats. Incorporated radioactivity was determined by liquid scintillation counting in a 2450 Microplate counter (Perkin Elmer, Waltham, MA).

### Statistical analysis

For statistical analysis, GraphPad Prism 7.03 (GraphPad Software, San Diego, CA) was used. Mean values and SEM are shown. The 95% confidence level was used and *P*-values were calculated with an unpaired two-tailed Student’s *t*-test or an unpaired two-tailed Welch’s *t*-test in the case of normally distributed data. Significance of not normally distributed data was either calculated with a paired two-tailed Wilcoxon matched-pairs signed-rank test or a two-tailed unpaired Mann–Whitney test. An unpaired analysis of variance (ANOVA) was used to analyze the differences among group means. *P*-value of *p* < 0.05 (*) was used as cutoff for significance.

## Results

### Generation and binding characteristics of NKG2D–CD16/CD3 fusion proteins

**Fig. 1:**
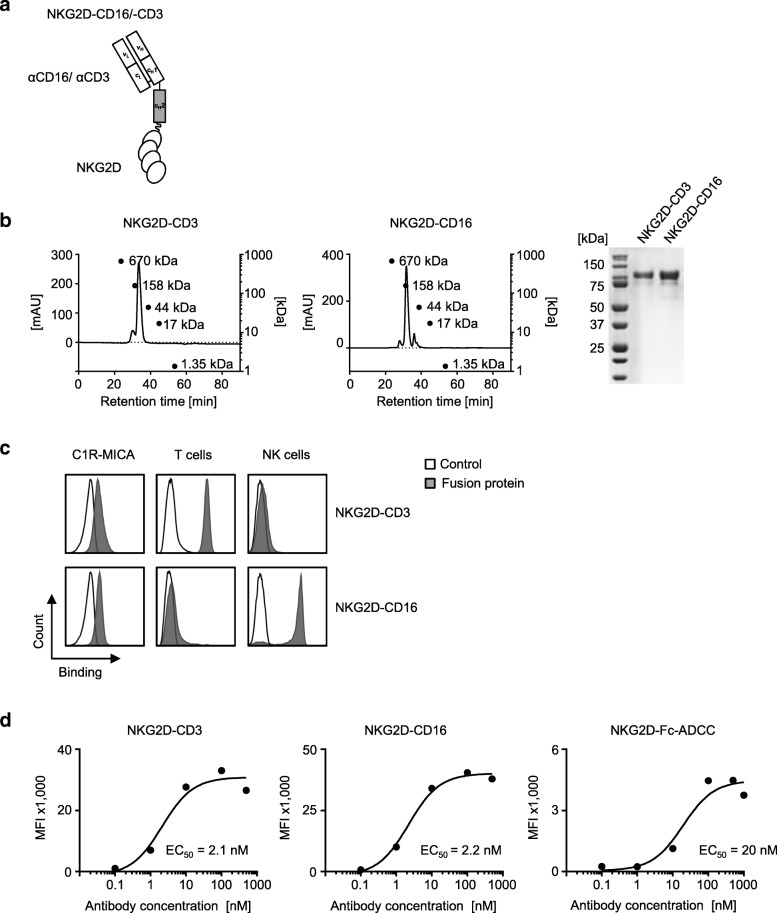
Generation and binding characteristics of the NKG2D–CD16/CD3 fusion proteins. **a** Schematic illustration of the NKG2D-CD3 and NKG2D-CD16 BFP consisting of the extracellular domain of NKG2D (F78-V216) fused with a CH_2_-linker to a CD3 or CD16 specific Fab-fragment. **b** Exemplary results of a size exclusion chromatography (left and middle panel) and a SDS PAGE (right panel) for both BFP. **c** Specific binding of the BFP was analyzed by flow cytometry using C1R-MICA transfectants and T cells and NK cells of healthy donors incubated with control (open peaks) or the BFP (shaded peaks) followed by an anti-human PE conjugate. **d** PBMC of healthy donors were incubated with the indicated concentrations of the BFP or NKG2D-Fc-ADCC followed by an anti-human PE conjugate and analyzed by flow cytometry. Exemplary data from one representative experiment of a total of three with similar results are shown. Mean fluorescence intensity (MFI) levels and EC_50_ were calculated using a sigmoidal dose-response curve with GraphPad Prism

As shown in Fig. 1a, our BFP consist of the extracellular domain of NKG2D (F78-V216) fused via a CH_2_-linker to either an anti-CD16 or an anti-CD3 Fab-fragment at the C-terminus. The constructs were then produced as described in the methods section. Size exclusion chromatography documented minimal aggregation tendencies. In SDS-PAGE, we observed single bands of about 110 kDa for NKG2D-CD16 and NKG2D-CD3. This confirmed that our constructs are stable and homogenous proteins with the expected molecular weight (Fig. 1b). Next we comparatively determined the binding capacities of our novel BFP by flow cytometry. Both constructs bound to C1R-MICA transfectants, while NKG2D-CD3 and NKG2D-CD16 specifically bound to CD3 and CD16 expressed by T cells and NK cells, respectively (Fig. 1c). Dose titration experiments revealed an EC_50_ of ~ 2 nM for binding of NKG2D-CD3 and NKG2D-CD16 to CD3 and CD16 expressed by T cells and NK cells, respectively. Binding of our previously described NKG2D-Fc-ADCC to FcγR on NK cells occurred with an EC_50_ of ~ 20 nM and thus with about ten-fold lower affinity compared to NKG2D-CD16, confirming the desired higher affinity of the CD3 and CD16 constructs (Fig. 1d).

### Modulation of NK and T cell reactivity by the anti-CD3/CD16 parts of the NKG2D fusion proteins

To characterize the potential of our constructs to stimulate effector cells, we first chose a setting independently of target cell binding. To this end, PBMC of healthy donors were cultured on immobilized NKG2D-Fc-ADCC, NKG2D-CD16, NKG2D-CD3 or the respective controls. Then NK cells and T cells within PBMC were selected by staining for CD56, CD3, CD4, CD8 and analyzed using flow cytometry for CD69 as early and CD25 as intermediate activation marker as well as CD107a as marker for degranulation after 24 h, 48 h and 4 h, respectively. In addition, IFN-γ secretion into culture supernatants as a further target cell independent marker for cellular activation was determined.

**Fig. 2:**
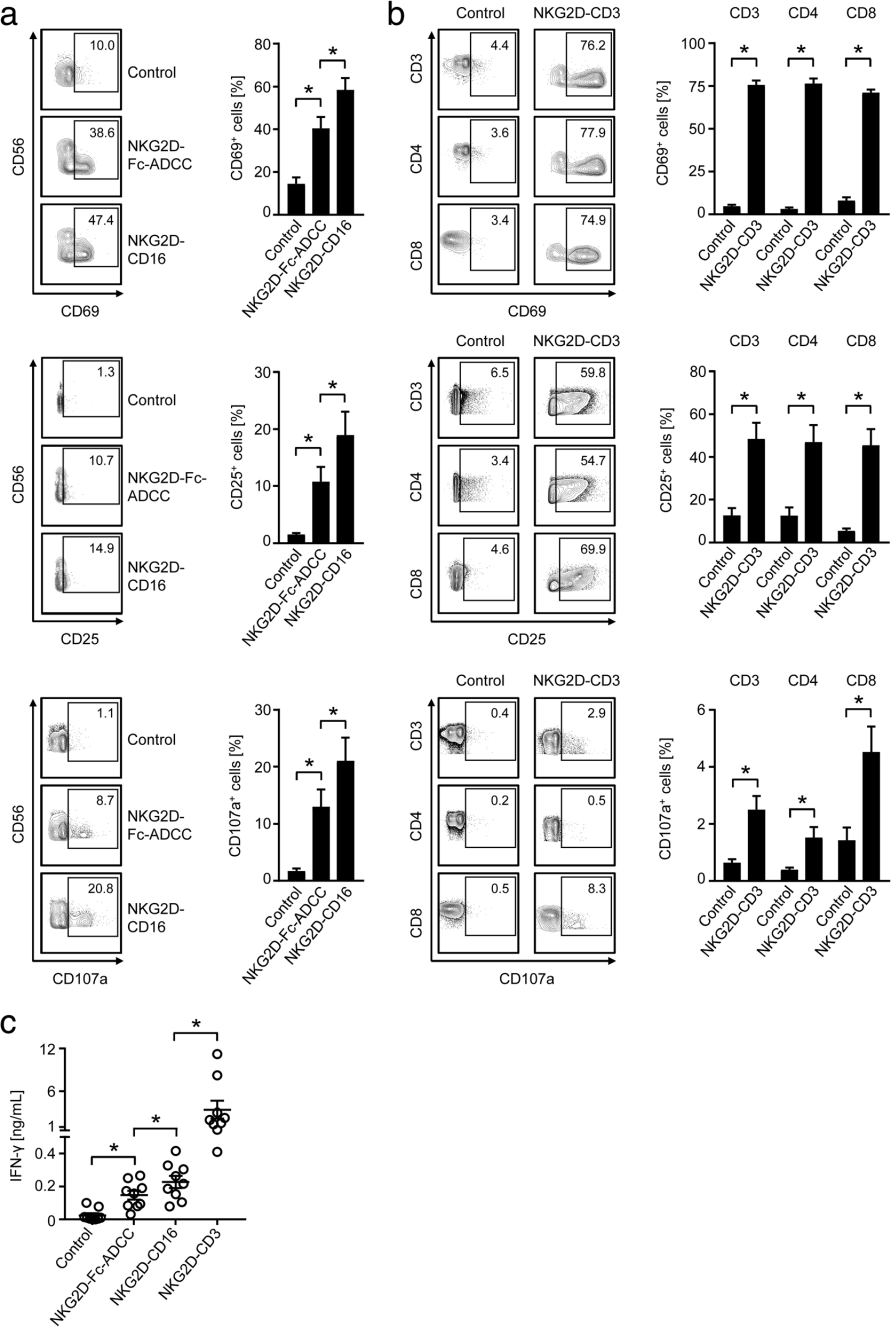
Modulation of NK and T cell reactivity by the anti-CD3/CD16 parts of the BFP. NKG2D-CD3, NKG2D-CD16, NKG2D-Fc-ADCC were immobilized to plastic as described in the methods section. Then PBMC of healthy donors were incubated on the immobilized fusion proteins or control. **a**, **b** Expression of CD69 and CD25 as markers for early and intermediate activation, and CD107a as marker for degranulation were determined after 24 h, 48 h and 4 h, respectively, on (**a**) NK cells after counterstaining for CD56^+^CD3^−^ or (**b**) T cells as identified by counterstaining for CD3, CD4 and CD8 by flow cytometry. Representative FACS plots of single experiments and combined data obtained with 8 different PBMC donors are shown (Mean ± SEM). **c** Supernatants of cultures were harvested after 4 h and IFN-γ levels were measured by ELISA (*n* = 10 different donors, Mean ± SEM)

When we compared the effects of our NKG2D-CD16 BFP to that of NKG2D-Fc-ADCC, we found that both constructs potently induced activation and degranulation of NK cells, with statistically significantly more pronounced effects mediated by NKG2D-CD16 (Fig. 2a). This is in line with its higher binding affinity to CD16. Profound T cell activation was observed upon exposure of PBMC to NKG2D-CD3 with comparable effects on CD4^+^ and CD8^+^ T cells. With regard to degranulation, effects were more pronounced on CD3^+^CD8^+^ T cells when compared to CD3^+^CD4^+^ T cells (Fig. 2b).

Analyses of IFN-γ secretion confirmed the higher stimulatory capacity of NKG2D-CD16 compared to NKG2D-Fc-ADCC; effects of both were by far exceed by the NKG2D-CD3 construct (Fig. 2c).

**Fig. 3:**
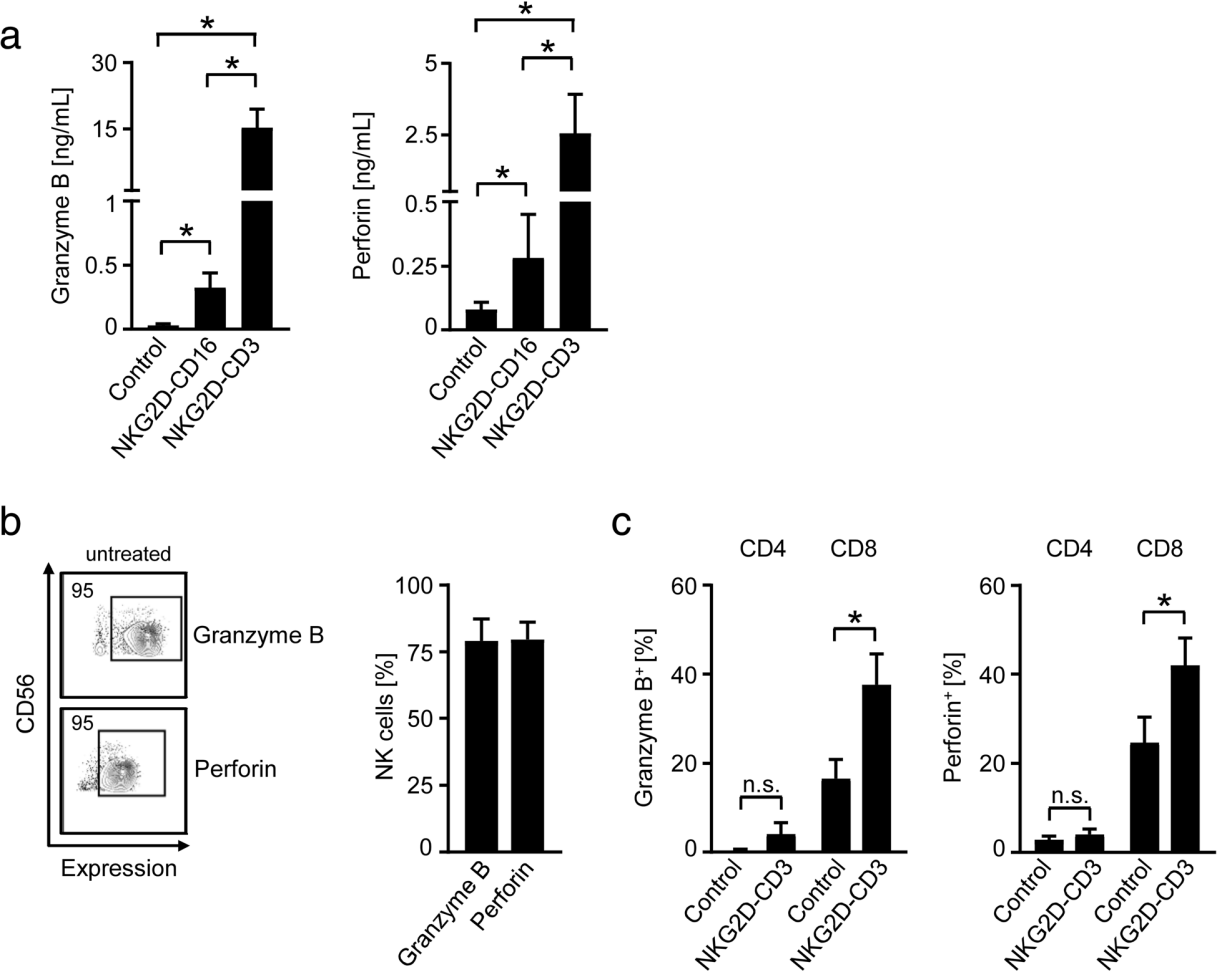
Modulation of effector cytotoxic capacity by BFP. NKG2D-CD3 and NKG2D-CD16 were immobilized to plastic as described in the methods section. Then PBMC of healthy donors were incubated on the immobilized fusion proteins or control. **a** Supernatants were analyzed for granzyme B and peforin levels after 24 h by Legendplex assays. Combined data obtained with 6 different PBMC donors are shown (Mean ± SEM). **b**, **c** Intracellular granzyme B and perforin expression was analyzed by flow cytometry. **b** Results obtained with untreated CD56^+^CD3^−^ NK cells (*n* = 3 different donors, Mean ± SEM); (**c**) results obtained with CD4^+^ and CD8^+^ T cells cultured in the presence of BFP or control (identified by counterstaining for CD3, CD4 and CD8, (*n* = 4, Mean ± SEM) are shown

To study how our BFP influenced the cytotoxic status of NK cells and T cells, we analyzed secretion of granzyme B and perforin after stimulation of PBMC for 24 h in the absence of target cells. Both NKG2D-CD16 and NKG2D-CD3 induced granzyme B and perforin secretion of PBMC with more pronounced effects observed upon exposure to NKG2D-CD3 (Fig. 3a). To allocate induction of granzyme B and perforin to the two effector cell subsets, we next analyzed NK cells and T cells after stimulation of PBMC with the two BFP by intracellular flow cytometric analyses. NK cells constitutively contained large amounts of granzyme B and perforin, and no further increase of intracellular expression upon treatment with NKG2D-CD16 was observed (Fig. 3b). In CD8^+^ T cells, NKG2D-CD3 potently increased the intracellular levels of granzyme B and perforin, whereas no or rather marginal effects were observed with CD4^+^ T cells (Fig. 3c).

### Modulation of NK and T cell reactivity against target cells

**Fig. 4:**
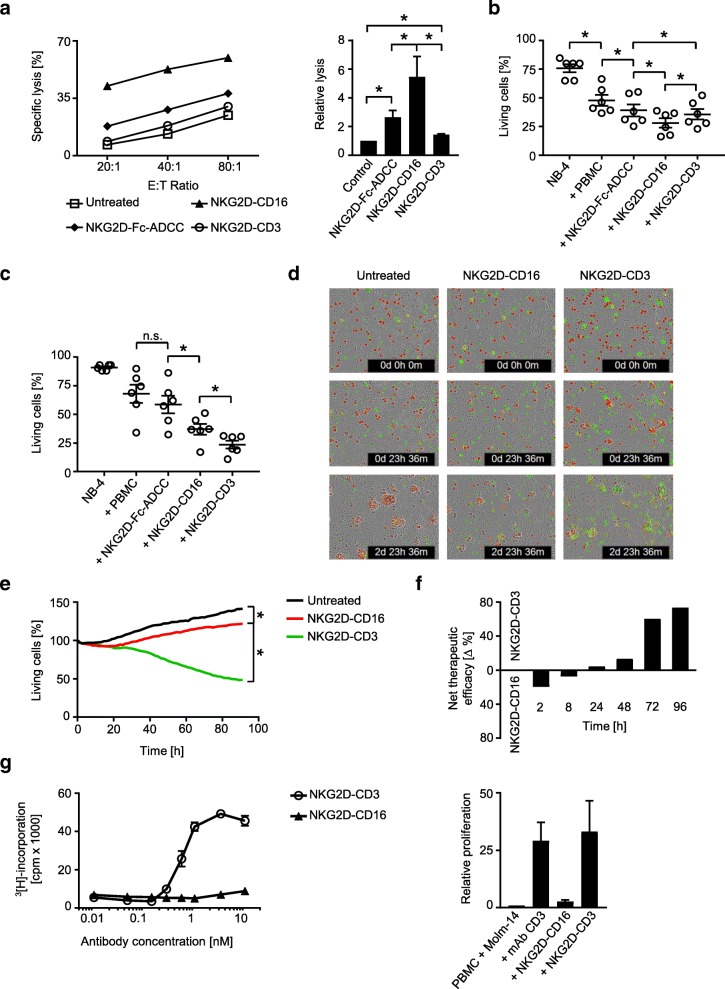
Effects of the BFP on target cell lysis and effector cell proliferation. **a**-**e** PBMC of healthy donors were incubated with leukemia cells treated with the indicated constructs (all 10 μg/mL) or left untreated. **a** Lysis of NB-4 leukemia cells was determined by 2 h cytotoxicity assays. Exemplary results (left panel) and combined data obtained with PBMC of 6 different healthy donors at an E:T ratio of 20:1 (right panel) are shown (Mean ± SEM). **b**, **c** Lysis of NB-4 leukemia cells was determined by flow cytometry based lysis assays at an E:T ratio of 20:1 after (**b**) 8 h and (**c**) 48 h using PBMC of 6 different donors (Mean ± SEM). **d**, **e** Cell death of THP-1 leukemia cells (E:T ratio 20:1) labelled with a red cell permeable dye was determined with an IncuCyte live cell imaging system using a Cytotox green cell death reagent over 96 h. Representative pictures at 0 h, 24 h and 72 h are shown (**d**) (THP-1 cells, red; dying cells, green; merged cells, yellow; magnification 4×). **f** Therapeutic efficacy of the two BFP was comparatively analyzed as follows: “% lysis NKG2D-CD16” – “% lysis NKG2D-CD3”. The net effect of the superior construct at the indicated time points is depicted. **g** PBMC of healthy donors were incubated with the indicated concentrations of the BFP in the presence of NKG2DL positive MOLM-14 cells for 72 h (E:T ratio 4:1). T cell proliferation was assessed by thymidine-uptake assays (left panel). Combined data obtained with PBMC of 4 different healthy donors at a concentration of 10 nM and with the parental CD3 mAb (UCHT1, 1.3 nM) as control (right panel) (Mean ± SEM)

Next we set out to determine the capacity of our BFP to induce NK and T cell reactivity against leukemia cells. To unravel potential differences of the induced immune responses according to stimulated effector cell type and thus comparison between the CD3 and CD16 stimulating constructs, we performed cytotoxicity assays with healthy PBMC and AML cell lines over various periods of time. Pronounced short-term killing (2 h) was observed in Europium cytotoxicity assays upon treatment with NKG2D-Fc-ADCC, and its effects were significantly exceeded by NKG2D-CD16 (Fig. 4a). NKG2D-CD3 mediated weak albeit significant effects in this experimental setting. After treatment for 8 h, flow cytometry-based assays revealed that NKG2D-CD16 still induced the most effective lysis with the effects of NKG2D-CD3 approximating (but still significantly differing from) that of NKG2D-Fc-ADCC (Fig. 4b). After 48 h, NKG2D-CD3 was found to induce superior leukemia cell killing compared to the NK cell activating constructs with NKG2D-CD16 still mediating profoundly stronger effects than NKG2D-Fc-ADCC (Fig. 4c). The superior effect of NKG2D-CD3 compared to NKG2D-CD16 after longer times of treatment was further confirmed by live cell imaging analyses over 96 h (Fig. 4d, e). Direct comparative analysis of the extent by which the two BFP mediated target cell lysis confirmed that NKG2D-CD16 was superior upon short term treatment, while NKG2D-CD3 mediated overall more potent effects manifesting at 24 h and thereafter (Fig. 4f). This likely can be attributed to the profound capacity of T cells to proliferate upon activation, as NKG2D-CD3 clearly induced T cell proliferation, while NK cells did not proliferate upon treatment with NKG2D-CD16. Notably, the effects of NKG2D-CD3 on T cell proliferation were comparable to that of the parental agonistic CD3 mAb as control (Fig. 4g). Combined treatment with the two BFP and thus parallel stimulation of NK cells and T cells resulted in higher target cell lysis rates compared to either construct alone (Additional file 1: Figure S1), and depletion of NK cells and T cells abrogated the effects of NKG2D-CD16 and NKG2D-CD3, respectively, confirming that the BFP specifically stimulate their respective effector cell population (Additional file 2: Figure S2). Notably, NKT cells did not contribute substantially to the BFP-induced target cell lysis, despite the fact that NKG2D-CD3 was found to potently activate this lymphocyte population. This can most likely be attributed to the rather low content of NKT cells within the employed PBMC preparations (Additional file 3: Figure S3). Addition of monensin in short term functional analyses completely abrogated the effects of the BFP without altering viability of the effector or target cells, which indicates that the activity of our constructs relies, at least in great part, on exocytosis of perforin/granzyme by the effector cells (Additional file 4: Figure S4). Together, these results confirm (i) the superior NK cell-stimulatory potential of NKG2D-CD16 as compared to NKG2D-Fc-ADCC and (ii) that stimulation of T cells induces more potent anti-leukemia effects compared to recruitment of NK cells, at least after longer times of treatment.

### Induction of NK and T cell reactivity against primary leukemia cells of AML patients

**Fig. 5:**
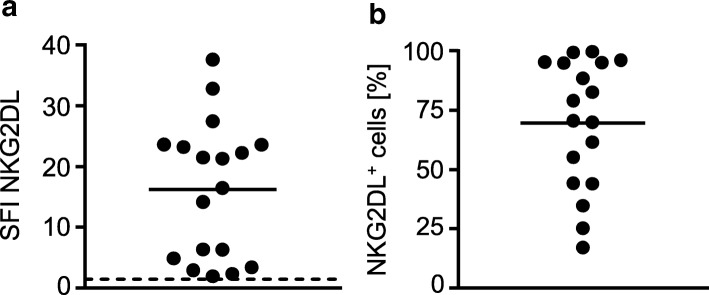
NKG2DL expression on primary AML samples. NKG2DL surface expression on primary AML cells of the patient samples employed in our study (*n* = 18) was analyzed by flow cytometry using a NKG2D-Fc chimera or isotype control (1 μg/mL) as described in the methods section. **a** SFI levels of NKG2DL expression of individual patients (threshold for surface positivity was defined as SFI ≥ 1.5 (dashed line), mean of the SFI levels (solid line)) are shown. **b** Percentage of NKG2DL^+^ AML cells within each sample is shown. Mean of percentage is depicted as solid line

As primary AML cells of different patients express varying patterns and levels of NKG2DL [6, 8, 13], we characterized the primary AML samples that were employed in the subsequent experiments. The clinical characteristics of each patient are given in Table 1. Determination of NKG2DL surface levels using a NKG2D-Fc chimera to simultaneously detect all different NKG2DL confirmed positivity in all utilized samples with SFI levels between 2.4 and 37.6 (Fig. 5a) and the percentage of NKG2DL^+^ AML blasts within individual samples ranging between 26% and ~ 100% (Fig. 5b).

**Table 1:**
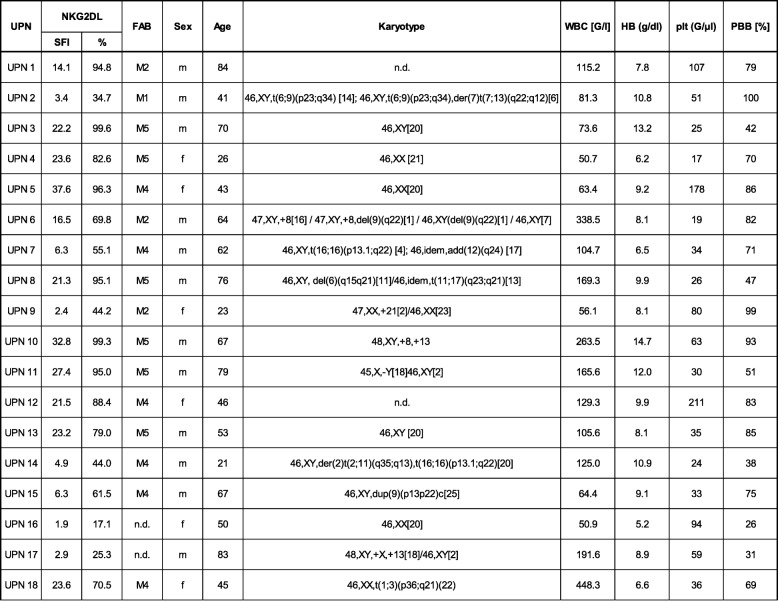
Patient characteristics and NKG2DL expression

UPN, uniform patient number; NKG2DL SFI, specific fluorescence index, % positive events; FAB, French-American-British classification; f, female; m, male; WBC, white blood count; HB, hemoglobin; plt, platelets; PBB, peripheral blood blasts among nucleated cells; n.d., not determined

**Fig. 6:**
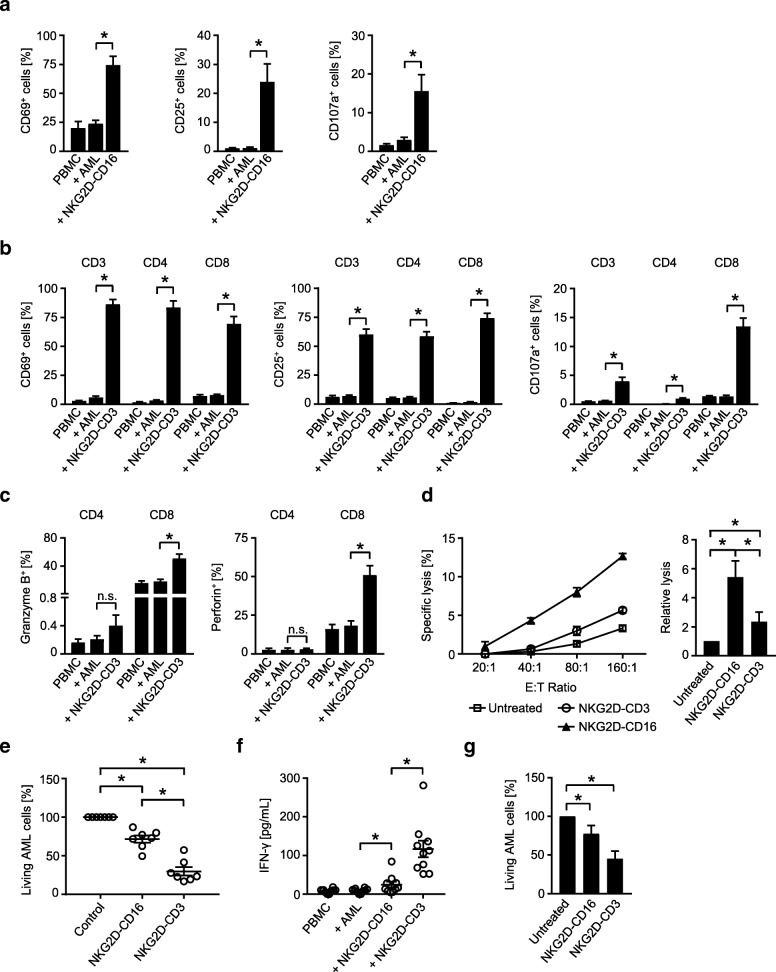
Anti-leukemia effects against patient leukemia cells. **a**-**f** Primary AML cells of patients were incubated with PBMC of healthy donors (E:T ratio 2.5:1) in the presence or absence of NKG2D-CD16, NKG2D-CD3 or control as indicated (all 10 μg/mL). **a**, **b** The expression of CD69 and CD25 as markers for early and intermediate activation, respectively, and CD107a as marker for degranulation were determined after 24 h, 48 h and 4 h, respectively, on (**a**) NK cells after counterstaining for CD56^+^CD3^−^ or (**b**) T cells as identified by counterstaining for CD3, CD4 and CD8 by flow cytometry. Combined data for NK cells obtained with 7 different PBMC donors (CD69 and CD107a) and 13 different PBMC donors for CD25 and with 8 different donors (CD69 and CD25) and 4 different donors (CD107a) for T cells are shown (Mean ± SEM). **c** Intracellular granzyme B and perforin expression in T cells (identified by counterstaining for CD3, CD4 and CD8) was analyzed by flow cytometry (n = 4, Mean ± SEM). **d** Lysis of leukemia cells was determined by 2 h cytotoxicity assays. Exemplary results obtained at the indicated E:T ratios (left) and combined data obtained with PBMC of 12 different healthy donors at an E:T ratio of 80:1 (right) are shown (Mean ± SEM). **e** Killing of leukemia cells was determined by flow cytometry based lysis assays at an E:T ratio of 5:1 after 96 h using PBMC of 7 different donors (Mean ± SEM). **f** Supernatants of cultures were harvested after 4 h and IFN-γ levels were measured by ELISA (*n* = 10 different donors, Mean ± SEM). **g** PBMC of AML patients with moderate blast counts (26–75%) were left untreated or incubated with BFP (10 μg/mL each) for 96 h. Then AML cell lysis by autologous NK cells and T cells was determined by flow cytometry. Combined data obtained with samples of 8 different AML patients are shown (Mean ± SEM)

As a first step, we determined the ability of our BFP to stimulate T and NK cell reactivity against the primary AML cells as targets. NKG2D-Fc-ADCC was not included in these analyses, as the previous experiments had already confirmed superior efficacy of NKG2D-CD16. Flow cytometry revealed that NKG2D-CD16 and NKG2D-CD3 potently stimulated activation (CD69 and CD25 upregulation) and degranulation (CD107a expression) of NK cells and T cells, respectively, contained in PBMC of healthy donors (Fig. 6a, b). With regard to degranulation, again more pronounced effects were observed with CD3^+^CD8^+^ compared to CD3^+^CD4^+^ T cells (Fig. 6b), which is in line with the results obtained in the analyses in the absence of target cells. In line with the results obtained by experiments in the absence of target cells described above, NKG2D-CD3 potently induced granzyme B and perforin in T cells (Fig. 6c). When the efficacy of the two BFP to induce leukemia cell lysis was comparatively analyzed, NKG2D-CD16 again mediated more potent effects in short term cytotoxicity assays (Fig. 6d), while profoundly stronger effects were observed with NKG2D-CD3 in analyses of long term lysis (Fig. 6e). The higher stimulatory capacity of NKG2D-CD3 compared to NKG2D-CD16 was also mirrored in analyses of IFN-γ secretion as second mechanism by which cytotoxic lymphocytes mediate antitumor reactivity (Fig. 6f).

In order to closely mirror the pathophysiological situation in leukemia patients, we next conducted ex vivo long term analyses with PBMC of patients with moderate AML cell counts (26–75% blasts) to study the effects of our BFP on NK and T cell-mediated lysis in an autologous system. Under these experimental conditions, profound effects of NKG2D-CD3 were observed, whereas NKG2D-CD16 mediated rather weak but significant effects, which is in line with our findings that NKG2D-CD3 mediates more potent effects than NKG2D-CD16 that require longer times of exposure to manifest (Fig. 6g).

## Discussion

The activating NKG2D receptor, which besides NK cells is expressed on different subsets of T cells [14, 15], interacts with multiple cell stress-induced, MHC class I-related ligands. These NKG2DL comprise, in humans, MICA, MICB and ULBP1–6 [16–18]. NKG2DL are selectively overexpressed on many cancer cells including leukemia, but largely absent on healthy tissues [5, 6]. They potently stimulate antitumor responses of cytotoxic lymphocytes in clear dependence of surface expression levels [19, 20]. This also holds true for AML cells, and substantial expression of at least one NKG2DL was observed in about 70% of AML patients [6]. In our study, samples were selected for functional analyses that mirrored the general prevalence and differing expression levels of NKG2DL in AML patients.

Due to the high immunostimulatory potential of tumor-expressed NKG2DL, multiple efforts aimed to modulate the NKG2D-NKG2DL system for cancer treatment. This comprised strategies to pharmacologically induce/enhance NKG2DL expression on malignant cells and the generation of constructs containing NKG2DL fused to tumor-targeting moieties [7, 13, 21–23]. We recently introduced fusion proteins consisting of the extracellular domain of NKG2D as targeting moiety, allowing for binding to all different NKG2DL, fused to a genetically optimized Fc-part (SDIE modification) for improved induction of ADCC by NK cells (NKG2D-Fc-ADCC). In both solid tumors and leukemia including AML, these constructs induced potent antitumor reactivity of NK cells [7, 8]. However, NKG2D-Fc-ADCC suffers from several drawbacks that we aimed to overcome in the present study: Besides binding to the immunostimulatory FcγRIII CD16 on NK cells, NKG2D-Fc-ADCC may also bind FcγRs expressed on non-cytotoxic cells (e.g., platelets and B cells), interact with FcγRs that do not trigger cytotoxicity (e.g. CD16b on granulocytes) and bind to inhibitory FcγR, which in turn may decrease its immunostimulatory potential. Maybe more importantly, T cells as second major cytotoxic lymphocyte compartment with their - compared to NK cells - higher effector potential cannot be stimulated by NKG2D-Fc-ADCC [24]. In fact, it is T cell-recruiting strategies that recently have revolutionized cancer treatment, at least in some tumor entities [25–28]. To overcome these shortcomings, we conceptualized NKG2D-CD16 and NKG2D-CD3 BFP with effector parts consisting of anti-CD16 and anti-CD3 Fab-fragments, the first allowing to bind CD16 specifically and with increased affinity, the second allowing for recruitment T cells.

Dose-titration experiments revealed about ten-fold higher affinity of NKG2D-CD16 to FcγRIII on NK cells compared to the FcγR binding of the optimized Fc-part of NKG2D-Fc-ADCC. The latter is also contained in many Fc-optimized mAbs like MOR00208 (anti-CD19 NCT01685021), Margetuximab (anti-Her2, NCT01828021), FLYSYN (anti-FLT3, ClinicalTrials.gov ID: NCT02789254), MEN1112 (anti-CD157, NCT02353143) and BI 836858 (anti-CD33, NCT02240706, NCT03013998) that are presently undergoing clinical evaluation, the latter three in AML. Functional studies in the absence and presence of leukemia cell lines and primary AML cells as targets and PBMC of healthy donors as effectors revealed the profound capacity of NKG2D-CD16 and NKG2D-CD3 to stimulate NK cells and T cells, respectively, as shown by analyses of activation, degranulation, granzyme B and perforin levels, IFN-γ secretion and target cell lysis in an allogeneic and notably also an autologous setting. In the latter, the efficacy of both BFP is certainly largely influenced by the highly varying percentages of both AML cells and effector cells in the different patient samples, and a potentially differing disease-induced impairment of patient NK cells and/or T cells may have further influenced the results. Overall, as expected based on its higher affinity to CD16, clearly more pronounced effects of NKG2D-CD16 were observed compared to NKG2D-Fc-ADCC. When analyzing the potential of NKG2D-CD3 to stimulate T cells, comparable results were observed in CD3^+^CD4^+^ and CD4^+^CD8^+^ T cell subsets with regard to activation. In contrast, significantly higher degranulation was observed within the cytotoxic CD8^+^ compared to CD4^+^ T cells. This is in line with data reported in previous studies that the higher cytotoxic capacity of CD8^+^ T cells is mirrored by increased degranulation [29, 30]. When comparing the therapeutic efficacy of the BFP and NKG2D-Fc-ADCC, NKG2D-CD16 mediated the most potent effects of all three on short-term lysis. NKG2D-CD3 required longer times to unravel its therapeutic potential, which after longer times of treatment, however, was clearly more pronounced when compared to the two NK cell activating constructs. This may be explained by our finding that NKG2D-CD3 potently induced T cell proliferation with a resulting increase of effector cells available to mediate anti-leukemic effects, which was not observed upon stimulation of NK cells. Notably, the direct comparison of the effects induced by our BFP over time and also analysis of effects obtained upon combined application would not have been possible when purified T cell and NK cell preparations would have been employed in separate experiments This underlines the advantage of our approach using whole PBMC as effector cells. Seemingly in contrast, IFN-γ release was more pronounced with NKG2D-CD3 compared to NKG2D-Fc-ADCC or NKG2D-CD16 already after treatment for 4 h. We hypothesize that this is due to the higher frequency of T cells (55–65%) compared to NK cells (10–25%) within the lymphocyte population contained in PBMC, which in turn results in increased release of preformed IFN-γ.

When weighing up NKG2D-CD16 and NKG2D-CD3 against each other, we speculate that the therapeutic efficacy and the potential side effects induced by the two constructs will closely correlate upon clinical application. NKG2D-CD16 may thus be better suited for treatment of frail or older patients which do not tolerate the potentially profound toxicity induced by T cell stimulatory approaches (for review [31–33]). It may be particularly suited in situations where disease burden is low, and, due to its improved stimulatory potential compared to conventional and also optimized Fc-parts, when the ability of NK cells to mediate ADCC is compromised, for example during immunosuppressive treatment after stem cell transplantation in AML [34–38]. NKG2D-CD3, in contrast, may constitute an optimal treatment for fit patients with higher leukemic burden. This notion is supported by the success of T cell mobilizing strategies that meanwhile have become a mainstay in oncological treatment: In solid tumors, blockade of inhibitory immune checkpoints like PD-1 und CTLA4 can induce remissions even in patients with progressed disease state [39–42]. Application of CART cells targeting CD19 is particularly effective in patients with lymphoid malignancies. Alike, the prototypical bsAb Blinatumomab (Amgen) in the so called BiTE-format targets CD19 and is meanwhile approved for the treatment of ALL [9, 10]. Both concepts are functionally closely related, but in theory bsAb provide the advantage of a standardized “of the shelf reagent” eliminating the laborious preparative work required for CART generation which results in delay of treatment (about 3 weeks) for patients and also high costs. In addition, the possibility to control anti-target activity via pharmacokinetics of bsAb application in contrast to CART cells allows for termination of activity. This advantage should also be taken into consideration when comparing our NKG2D-CD3 construct with NKG2D-CAR-expressing T cells that have been described previously and presently undergo clinical evaluation (NCT02203825) [43].

A common drawback of available CART cells and Blinatumomab are the in part severe side effects occurring with either strategy. In case of bsAbs constructed as BiTE molecules like Blinatumomab, one causal problem is their aggregation tendency, which results in “*off target*” activation of T cells. An even more important problem that affects both CART and bsAbs targeting CD19 is that this antigen is not only expressed on malignant cells, but also on healthy B cells. This results in deleterious non-tumor restricted “*on target-off tumor*” activation of the T cell system.

In addition, CD19 is expressed mainly in lymphoid leukemias and thus only a minor part of all acute leukemias. No immunotherapeutic treatment option is presently established for patients with AML, which constitutes the majority of acute leukemia cases in adults. Beyond mAbs like those mentioned above, bsAbs and CART cells directed to myeloid antigens are in development [25–27, 44, 45], but at least some of the above mentioned drawbacks hold true for the latter compounds. For example, when targeting CD33, which is abundantly expressed on healthy myeloid cells, even more “*on target-off tumor*” toxicity than with Blinatumomab is to be expected. Targeting NKG2DL as achieved by our BFP not only allows to establish a treatment option for AML patients, but also holds promise to reduce side effects due to the absence of NKG2DL on healthy cells and thus clearly leukemia cell-restricted target antigen expression. It remains to be determined how the activity of our BFP is affected by soluble NKG2DL that are present at elevated levels in sera of cancer patients including AML [6, 46, 47]. Soluble NKG2DL might compromise binding of the BFP to their surface-expressed counterparts and thus impair activity. However, in theory this could be overcome by application of sufficient amounts of the BFP, and at the same time facilitate neutralization of the detrimental effects of soluble NKG2DL that compromise immune surveillance in cancer patients [5]. Certainly, substantial further work is required to clarify this and other open questions before patients can be treated with our BFP. Nevertheless, the conceptualized NKG2D-CD16 or NKG2D-CD3 constructs that allow for a treatment that can be tailored to the specific patient condition and disease state/burden, in our view constitute a promising option for personalized immunotherapy of AML.

## Additional files

Additional file 1: **Figure S1.** Combination of NKG2D-CD3 & NKG2D-CD16 in leukemia cell lysis (PDF 67 kb)

Additional file 2: **Figure S2.** Induction of leukemia cell lysis by BFP after depletion of effector cells (PDF 60 kb)

Additional file 3: **Figure S3.** Effects of NKG2D-CD3 on NKT cells (PDF 66 kb)

Additional file 4: **Figure S4.** Influence of blocking granule exocytosis on BFP-induced effector cell functionality (PDF 58 kb)

## Abbreviations

7-AAD: 7-aminoactinomycin D
Ab: Antibody
ADCC: Antibody dependant cellular cytotoxicity
ALL: Acute lymphoblastic leukemia
AML: Acute myeloid leukemia
BFP: Bispecific fusion protein
BiTE: Bispecific T cell engager
bsAb: Bispecific antibody
CAR: Chimeric antigen receptor
E:T: Effector to target
ELISA: Enzyme-linked immunosorbent assay
EU: Endotoxin
FACS: Fluorescence-activated cell sorting
FcγR: Fc-gamma-receptor
IFN-γ: Interferon-γ
mAb: monoclonal antibody
MFI: Mean fluorescence intensity
MIC: MHC class I chain-related
NKG2D: Natural killer group 2D
NKG2DL: NKG2D ligand
PBMC: Peripheral blood mononuclear cell
PE: Phycoerythrin
PI: Propidium iodide
SDIE: Amino acid modifications S239D and I332E
SDS-PAGE: Sodium dodecyl sulfate polyacrylamide gel electrophoresis
SEM: Standard error of the mean
SFI: Specific fluorescence intensity
TCR: T cell receptor
ULBP: UL16 binding protein
