# Supplementary material for: Bispecific NKG2D-CD3 and NKG2D-CD16 fusion proteins for induction of NK and T cell reactivity against acute myeloid leukemia
Source: J Immunother Cancer. 2019 May 29;7:143. doi: 10.1186/s40425-019-0606-0 (PMC6542021; doi:10.1186/s40425-019-0606-0)
Supplement: Supplementary file 1 — Figure S1. Combination of NKG2D-CD3 & NKG2D-CD16 in leukemia cell lysis (PDF 67 kb) [file 40425_2019_606_MOESM1_ESM.pdf]

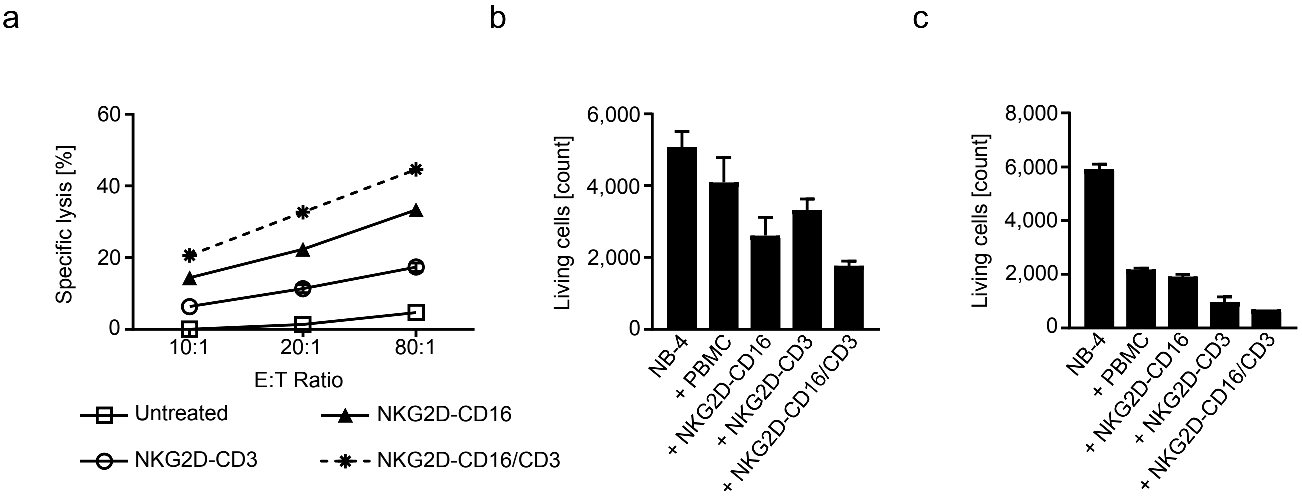

Supplementary Figure 1: Combination of NKG2D-CD3 & NKG2D-CD16 in leukemia cell lysis

PBMC of healthy donors were incubated with NB-4 leukemia cells and treated with the indicated constructs (all 10µg/mL) or left untreated.

(a) Lysis of leukemia cells was determined by 2h cytotoxicity assays. Exemplary results of three independent experiments with similar results are shown.

(b, c) Lysis of leukemia cells was determined by flow cytometry based lysis assays at an E:T ratio of 20:1 after (b) 8h and (c) 48h. Exemplary results of three independent experiments with similar results are shown.
