# Supplementary material for: Bispecific NKG2D-CD3 and NKG2D-CD16 fusion proteins for induction of NK and T cell reactivity against acute myeloid leukemia
Source: J Immunother Cancer. 2019 May 29;7:143. doi: 10.1186/s40425-019-0606-0 (PMC6542021; doi:10.1186/s40425-019-0606-0)
Supplement: Supplementary file 2 — Figure S2. Induction of leukemia cell lysis by BFP after depletion of effector cells (PDF 60 kb) [file 40425_2019_606_MOESM2_ESM.pdf]

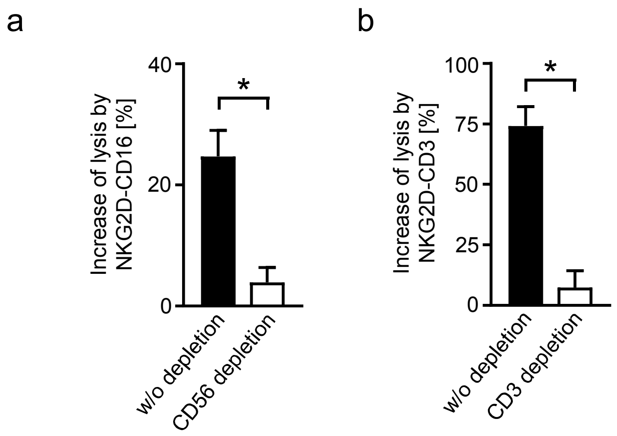

Supplementary Figure 2: Induction of leukemia cell lysis by BFP after depletion of effector cells

PBMC of healthy donors were incubated with NB-4 leukemia cells in the presence of 10µg/mL NKG2D-CD16 (a) or NKG2D-CD3 (b). Where indicated, CD56+ and CD3+ cells had been depleted by magnetic bead separation with CD56 and CD3 Microbeads (MACS Miltenyi), respectively, prior to culture according to the manufacturer's instructions.

(a) Depicted is the NKG2D-CD16 induced increase in leukemia cell lysis as determined by 2h cytotoxicity assays. Lysis rates obtained in the absence of NKG2D-CD16 were set to 100% and results obtained with NKG2D-CD16 were normalized accordingly. Combined data obtained with three different PBMC donors (E:T ratio 40:1) are shown for whole and corresponding CD56 depleted PBMC (Mean ± SEM).

(b) NKG2D-CD3 induced increase in leukemia cell lysis as determined by flow cytometry based lysis assays after 48h at an E:T ratio of 20:1. Lysis rates obtained in the absence of NKG2D-CD3 were set to 100% and results obtained with NKG2D-CD3 were normalized accordingly. Combined data obtained with three different PBMC donors are shown for whole and corresponding CD3 depleted PBMC (Mean ± SEM).
