# Supplementary material for: Bispecific NKG2D-CD3 and NKG2D-CD16 fusion proteins for induction of NK and T cell reactivity against acute myeloid leukemia
Source: J Immunother Cancer. 2019 May 29;7:143. doi: 10.1186/s40425-019-0606-0 (PMC6542021; doi:10.1186/s40425-019-0606-0)
Supplement: Supplementary file 3 — Figure S3. Effects of NKG2D-CD3 on NKT cells (PDF 66 kb) [file 40425_2019_606_MOESM3_ESM.pdf]

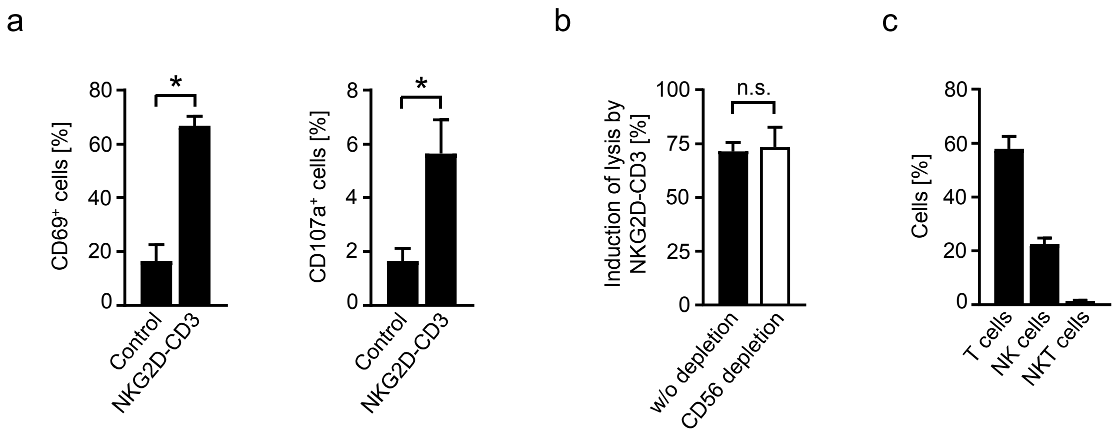

Supplementary Figure 3: Effects of NKG2D-CD3 on NKT cells

(a) NKG2D-CD3 or control was immobilized to plastic as described in the methods section. Then PBMC of healthy donors were incubated on the immobilized fusion proteins or control and CD69 and CD107a as markers for activation and degranulation were determined by flow cytometry after 24h and 4h, respectively. NKT cells were identified by counterstaining for $CD56^{+}CD3^{+}$. Combined data obtained with 4 different PBMC donors are shown (Mean ± SEM).

(b) PBMC of healthy donors were incubated with NB-4 leukemia cells in the presence or absence of 10µg/mL NKG2D-CD3. Where indicated, $CD56^{+}$ cells had been depleted by magnetic bead separation with CD56 Microbeads (MACS Miltenyi) prior to culture according to the manufacturer's instructions. The NKG2D-CD3 induced increase in leukemia cell lysis as determined by flow cytometry based lysis assays at after 48h an E:T ratio of 20:1 is depicted. Lysis rates obtained in the absence of NKG2D-CD3 were set to 100% and results obtained with NKG2D-CD3 were normalized accordingly. Combined data obtained with three different PBMC donors are shown for whole and corresponding CD56 depleted PBMC (Mean ± SEM).

(c) The percentage of $CD3^{+}$ T cells, $CD56^{+}CD3^{-}$ NK cells and $CD56^{+}CD3^{+}$ NKT cells contained in the utilized PBMC preparations was determined by flow cytometry (Mean ± SEM).

Märklin et al., Supplementary Figure 3
