# Supplementary material for: Bispecific NKG2D-CD3 and NKG2D-CD16 fusion proteins for induction of NK and T cell reactivity against acute myeloid leukemia
Source: J Immunother Cancer. 2019 May 29;7:143. doi: 10.1186/s40425-019-0606-0 (PMC6542021; doi:10.1186/s40425-019-0606-0)
Supplement: Supplementary file 4 — Figure S4. Influence of blocking granule exocytosis on BFP-induced effector cell functionality (PDF 58 kb) [file 40425_2019_606_MOESM4_ESM.pdf]

a

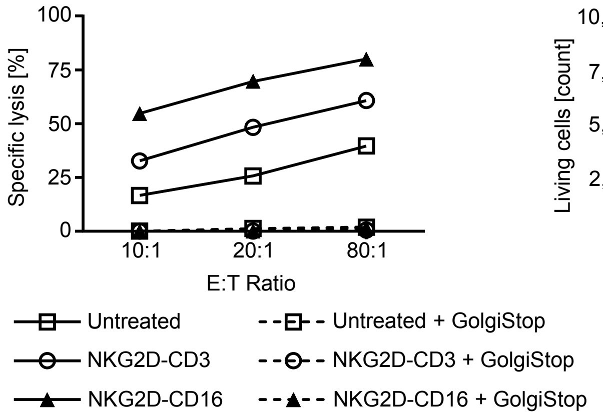

b

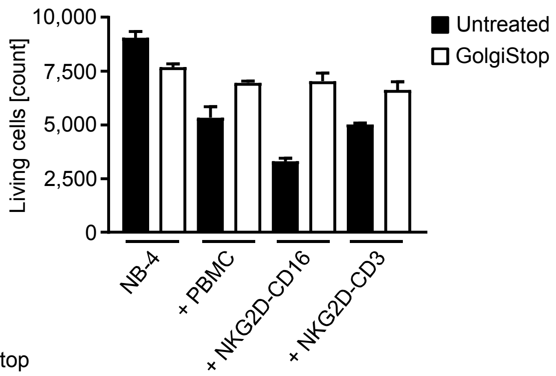

c

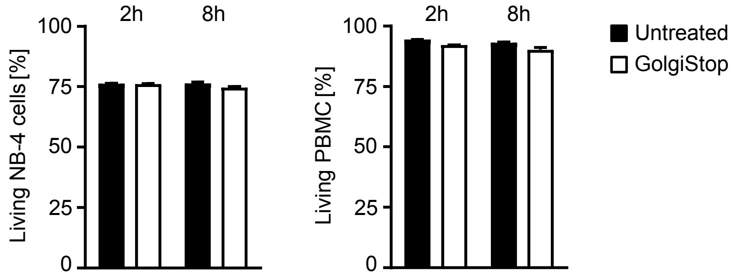

Supplementary Figure 4: Influence of blocking granule exocytosis on BFP-induced effector cell functionality

(a-b) PBMC of healthy donors were pretreated for 30min with monensin (GolgiStop, BD Biosciences) according to manufacturer’s instructions. Then NB-4 leukemia cells and the indicated constructs (all 10µg/mL) were added.

(a) Lysis of leukemia cells was determined by 2h cytotoxicity assays. Exemplary results of three independent experiments with similar results are shown.

(b) Lysis of leukemia cells at an E:T ratio of 20:1 was determined by flow cytometry based lysis assays after 8h. Exemplary results of three independent experiments with similar results are shown.

(c) Leukemia cells (left) or PBMC (right) were cultured for 2h or 8h with monensin according to manufacturer’s instructions. Then the percentage of living cells was analyzed by flow cytometry using PI. Exemplary results of three independent experiments with similar results are shown.
